# Regulation of the Fruit-Specific PEP Carboxylase *SlPPC2* Promoter at Early Stages of Tomato Fruit Development

**DOI:** 10.1371/journal.pone.0036795

**Published:** 2012-05-17

**Authors:** Carine Guillet, Mourad A. M. Aboul-Soud, Aline Le Menn, Nicolas Viron, Anne Pribat, Véronique Germain, Daniel Just, Pierre Baldet, Patrick Rousselle, Martine Lemaire-Chamley, Christophe Rothan

**Affiliations:** 1 Unité Mixte de Recherche 1332 Biologie du Fruit et Pathologie, Institut National de la Recherche Agronomique, Villenave d’Ornon, France; 2 Unité Mixte de Recherche 1332 Biologie du Fruit et Pathologie, Université Bordeaux, Villenave d’Ornon, France; 3 Biochemistry Department, Faculty of Agriculture, Cairo University, Giza, Egypt; 4 Chair of Medical and Molecular Genetics Research, Department of Clinical Laboratory Sciences, College of Applied Medical Sciences, King Saud University, Riyadh, Saudi Arabia; 5 Unité de Génétique et d’Amélioration des Fruits et Légumes, Institut National de la Recherche Agronomique, Montfavet, France; Friedrich-Alexander-University Erlangen-Nurenberg, Germany

## Abstract

The *SlPPC2* phospho*enol*pyruvate carboxylase (PEPC; EC 4.1.1.31) gene from tomato (*Solanum lycopersicum*) is differentially and specifically expressed in expanding tissues of developing tomato fruit. We recently showed that a 1966 bp DNA fragment located upstream of the ATG codon of the *SlPPC2* gene (GenBank AJ313434) confers appropriate fruit-specificity in transgenic tomato. In this study, we further investigated the regulation of the *SlPPC2* promoter gene by analysing the *SlPPC2 cis*-regulating region fused to either the firefly luciferase (LUC) or the β-glucuronidase (*GUS*) reporter gene, using stable genetic transformation and biolistic transient expression assays in the fruit. Biolistic analyses of 5′ *SlPPC2* promoter deletions fused to LUC in fruits at the 8^th^ day after anthesis revealed that positive regulatory regions are mostly located in the distal region of the promoter. In addition, a 5′ UTR leader intron present in the 1966 bp fragment contributes to the proper temporal regulation of LUC activity during fruit development. Interestingly, the *SlPPC2* promoter responds to hormones (ethylene) and metabolites (sugars) regulating fruit growth and metabolism. When tested by transient expression assays, the chimeric promoter:LUC fusion constructs allowed gene expression in both fruit and leaf, suggesting that integration into the chromatin is required for fruit-specificity. These results clearly demonstrate that *SlPPC2* gene is under tight transcriptional regulation in the developing fruit and that its promoter can be employed to drive transgene expression specifically during the cell expansion stage of tomato fruit. Taken together, the *SlPPC2* promoter offers great potential as a candidate for driving transgene expression specifically in developing tomato fruit from various tomato cultivars.

## Introduction

Tomato (*Solanum lycopersicum*) is currently the plant model for the study of fleshy fruit development. Several national and international initiatives such as the SOL consortium have contributed to develop new genomic resources in tomato, including the sequencing of tomato genome, the generation of large scale EST and full-length cDNA collections [1], [2] and the expression profiling of developing fruit tissues [3]–[6]. Mining available tomato genomic resources has now produced a wealth of candidate genes with potential roles in the regulation of early fruit development and metabolism [7]. One of the methods of choice for analysing their functional role in the fruit or for bioengineering fruit quality is the generation of stable transgenic lines in which the expression of the candidate gene is specifically modulated in the tissue or at the developmental stage of interest 8,9. In this context, the use of fruit-specific promoters instead of constitutive promoters which may trigger non-specific alterations at whole plant level is usually preferable. To this end, new tomato transformation vectors integrating fruit-specific promoters have recently been crafted for the study of *Solanaceae* genes [10], [11]. One of these tool kits includes the promoter from the *SlPPC2* tomato fruit-specific carboxylase gene previously isolated in our group [12]. In the MicroTom cultivar, the *SlPPC2* promoter can be used to direct the mis-expression or silencing of genes-of-interest specifically in the expanding cells from developing tomato fruit [10]. These findings open new ways for the study of the cell expansion phase, which follows the cell division stage and precedes the onset of fruit ripening [13]. This period is crucial not only for fruit growth but also for the acquisition of other fleshy fruit attributes such as the accumulation of water, organic acids, starch and secondary metabolites of high nutritional and sensorial value. As an example of the use of the *SlPPC2* promoter, the specific modulation of the cell cycle-related CDK inhibitor KRP in enlarging tomato fruit cells recently led to the demonstration that growth of tomato fruit cells could be uncoupled from cell ploidy level [14]. Such original result was not achieved previously by using the *CaMV35S* promoter to direct the expression of the *CSCS52* endoreduplication-related gene [15], thus demonstrating the power of this approach. Additional insights into the regulation of the *SlPPC2* promoter are now needed to delineate more precisely its mode of action in the various cell types of the fruit pericarp.

The transcripts from the *SlPPC2* gene encoding a fruit-specific phospho*enol*pyruvate carboxylase (PEPC; EC 4.1.1.31) are among the most abundant transcripts found in expanding tomato fruit [12]. One of the functions fulfilled by PEPC is the replenishment of the TCA cycle with oxaloacetate by catalyzing the PEP to oxaloacetate conversion [16]. PEPC appears therefore as a key enzyme in the synthesis of malic and citric acids [16], the two major organic acids accumulated in most fleshy fruits. Regulation of fruit PEPCs is however poorly known. In addition to the tight control of PEPC activity exerted at post-translational level [17], [18], [19], evidence for coarse transcriptional and translational control of PEPC has been presented [16], [20]–[24]. In the C4-type maize, transcription of PEPC has been shown to be regulated by development, light, glucose and acetate [25]. In addition, elements of the tissue-specific and light-regulated control of expression of C4 PEPCs have been identified [24], [26], [27]. In contrast, much less data are available on non-photosynthetic PEPCs, which include the *SlPPC2* fruit PEPC [12], though recent advances have shed new light on their regulation and functions [28].

To gain further insights onto the transcriptional regulation of *SlPPC2* during the cell expansion stage, and to evaluate the potential use of *SlPPC2* promoter for driving gene expression in various genetic or environmental contexts in tomato, we studied the regulation of *SlPPC2* promoter in the early developing fruit. Combination of transient expression assays by particle bombardment of pericarp discs and of studies on transgenic tomato plants confirmed that the *SlPPC2* promoter is able to confer a proper developmental regulation in the fruit. Strikingly, the fruit-specific expression of *SlPPC2* promoter, observed in stable transgenic lines, was lost in transient expression assays, suggesting the need for chromatin integration for appropriate transcriptional regulation in the plant. This study also emphasizes the role of the leader intron located in the 5′UTR of the gene as a negative regulator of *SlPPC2* and highlights the possible role of hormones (ethylene) and metabolites (sugars) in its regulation.

## Materials and Methods

### Ethics Statement

N/A.

### Plant Material

Transgenic tomato plants (*Solanum lycopersicum*, cv. «Ferum») expressing *GUS* reporter gene under the control of *CaMV35S* or *SlPPC2* promoters were grown in greenhouse as previously described [29], [30]. Plant tissues (seedling, leaflet and flower) and fruits were collected at the indicated stages of development for GUS staining. Biolistic transient expression assays were carried out using cherry tomato fruits (*Solanum lycopersicum*, cv. «WVa 106») cultivated under growth chamber conditions: cycles of 15 h (25°C) day and 9 h (20°C) night; light intensity of 400 µmole m^−2^ s^−1^. Number of inflorescence was limited to 3 per plant. Flowers were tagged on the plant at anthesis and fruits were harvested at the indicated stage, from 6 to 35 days after anthesis (daa), according to age and diameter. The mature green (30 daa) and orange (35 daa) fruits were further selected according to color. Leaflets of young leaves were collected from the same plants.

### Isolation of SlPPC2 and Analysis of its Promoter Sequence

A genomic *SlPPC2* clone with an insert size of 15 kb was obtained after screening a λ EMBL-3 tomato genomic library (var. «VFN8») (Clontech) with a 566-bp fragment PCR-amplified from the *SlPPC2* cDNA clone [12] and sequenced (GenBank AJ313434). The genomic *SlPPC2* insert isolated contained the entire coding region (5470 bp) plus 5 kb of sequence upstream the coding region and 4 kb downstream. The transcription start point of the *SlPPC2* gene was determined by primer extension analysis using a reverse primer 5PEPC2AC (5′-GAACCCAGAGATGAAGAAAGG-3′) located 57 to 78 bp upstream of the translation initiation ATG codon. The extension reaction was performed at 37°C for 90 min with 100 units of M-MLV reverse transcriptase (Invitrogen) and 50 µM each of dCTP, dTTP and dGTP, and 50 µM of α-[^33^P]-ATP. The resulting DNA fragment was analyzed on a 6% polyacrylamide gel and was mapped by comparison to a sequence ladder produced from the *SlPPC2* promoter using 5PEPC2AC primer to determine transcription start point. The *SlPPC2* promoter was analyzed using PLACE [31], PlantCARE [32] and MAR Finder [33].

### Reporter Gene Constructs for Biolistic Assays

For biolistic transient expression assays, the plasmid pRTL2-GUS consisting of the *CAMV 35S* promoter upstream of the tobacco etch virus leader fused to the *GUSA* gene of *E. coli* (here referred to as 35S-GUS) was used as a reference construct. A series of five promoter:LUC fusion plasmids were prepared for gene-expression analysis of the *SlPPC2* promoter. The promoter fragments (−1528 to +439 [pPPC2pro1:LUC], −980 to +439 [pPPC2pro2:LUC], −430 to +439 [pPPC2pro3:LUC], −70 to +439 [pPPC2pro4:LUC] and −1528 to +195 [pPPC2pro5:LUC]) were PCR-amplified from the pCR-Script-SlPPC2 plasmid as template using the *SlPPC2*-specific primers designed with either a *Sac*I or a *Not*I site at their 5′ end. They were further cloned into *Sac*I*/Not*I sites of pGreen 0000SK LR [34] and sequenced. The LUC gene-nos 3′ terminator cassette from RBCS2-LUC was excised by *Nhe*I and *Eco*RI and ligated using *Xba*I and *EcoR*I sites into the five pGreen 0000SK LR plasmids containing the promoter fragments. All constructs were confirmed by sequencing.

### Reporter Gene Construct and Generation of Tomato Transgenic Plants

A −1528 to +439 bp 5′ fragment relative to the transcription start site was cloned into the plant transformation vector pGreen 2 K vector at *Xho*I (5′) and *Eco*RI (3′) sites with GUS as reporter gene. This *SlPPC2* promoteur:GUS construct was introduced into «Ferum» tomato (a medium fruit-sized greenhouse type cultivar) by *Agrobacterium tumefaciens* strain GV3101 according to a published protocol [35]. Regenerated plantlets were further checked for ploidy level by flow-cytometry analysis and polyploid plants were discarded. Up to twelve independent plants were generated and screened for GUS staining. Control plants corresponding to plants transformed with 35S-GUS or an empty vector were analyzed in parallel. Results presented are from a representative GUS staining experiment. Cherry «WVa106» cultivar was transformed with a *SlPPC2* promoter:GFP-GUS fusion generated by cloning a 1972 bp *SlPPC2* promoter fragment (including the 5′UTR and leader intron) into the pKGWFS7 vector [36].

### Particle Bombardment

Experimental conditions were essentially as previously defined [37] for biolistic transient-expression assays in developing tomato fruit, with modifications. Each tomato fruit was cut into three thin slices (0.5 to 1.0 mm thickness) and soaked for 5 min in CPW 12 [38] supplemented with 12% (w/v) mannitol, 20 mM MES, pH 6.0. Young leaves were cut into pieces of approximately 1 cm^2^. When indicated, sugars (sucrose [5 to 100 mM], 3-0-methylglucose [50 mM], 2-deoxyglucose (50 mM), fructose (50 mM), glucose (50 mM)] or hormones [GA3 (5 µM), 2,4-D (0.5 to 500 µM), Kinetin (5 µM), ABA (50 µM), ACC (20 and 200 µM)] were added to the CPW 12 medium. In the experiments designed to inhibit ethylene action, fruit tissues were incubated for 2.5 min before osmotic treatment with 4 M silver thiosulfate (AgTS) or with 4 M sodium thiosulfate (control) as previously described [39]. All compounds were dissolved in water or dimethyl sulfoxide and the aqueous solutions were filter-sterilized before use.

Tungsten particles (7 mg, 1.1 µm diameter, Bio-Rad) were coated with either 15 µg reporter plasmid or a 1∶1 ratio of reporter and reference plasmid (15 µg each) in order to obtain 10 cartridges for the helium-driven Gene Gun Helios System (Bio-Rad). Each fruit slice was placed on plate and bombarded with DNA-coated tungsten particles from one cartridge. The Gene Gun was perpendicular to the fruit or leaf surface, its spacer touched the target area and a helium pressure of 210 *psi* was used. Fruit slices were bombarded a second time after flipping the slices on the plate. The bombarded fruit tissues were placed on 0.8% agar in CPW 4 (4% [w/v] mannitol) supplemented or not with the various hormones and metabolites as indicated above and were incubated for 20 h under growth chamber conditions (22°C, light). The bombarded leaves were placed on 0.8% agar in H_2_O and incubated for 20 h under growth chamber conditions (22°C, light). These conditions differ from those previously described [37] and were found to be the best adapted to the plant material (cherry tomato fruit, cv. «WVa106») and the biolistic system (BioRad Gene Gun) used. For each construct analyzed and for a given tissue or developmental stage, 9 to 15 tissue samples were independently bombarded and analyzed for Luciferase and GUS activities.

### Luciferase and GUS Assays

For biolistic transient expression assays, the fruit or leaf discs were weighed, frozen in liquid nitrogen, ground in a mortar, and homogenized with a 1 ml Tenbroeck Tissue Grinder (Wheaton Millville) in lysis buffer (0.3 M Tris-phosphate pH 7.8, 2 mM dithiotreitol, 2 mM diaminocyclohexane tetracetic acid, 10% glycerol, 1% Triton ×-100) [40], using 2 mL buffer g^−1^ plant tissue. The extract was cleared by centrifugation (15 000×g for 10 min). Protein concentration was determined with the Coomassie plus protein assay reagent (Pierce) adapted for the MR5000 microplate reader (Dynatech) using BSA as a standard. Luciferase activity was determined immediately after extract preparation using the Promega Luciferase Assay System (Promega) as previously described [41]. Light emission was measured for 1 min in a 1254 Luminova luminometer (Bio-Orbit Oy). Statistical comparisons between results within a given experiment were made using a Student’s *t*-test. All differences were significant to at least a value of *P*<5%. GUS activity was determined by adding 20 µL of supernatant to 2 mL GUS buffer (50 mM NaH_2_PO_4_ pH7, 10 mM Na_2_EDTA, 10 mM β-mercaptoethanol, 0.1% Sarcosyl, 0.1% Triton ×100) containing 0.75 g/mL of 4-methyl umbelliferyl β-D-glucuronide (MUG). After incubation at 37°C, 400 µL of reaction mixture was collected at 0, 30 and 90 min and mixed with 2 mL stop buffer (200 mM Na_2_CO_3_). Fluorescence was measured at 455 nm after excitation at 365 nm (Hitachi spectrofluorimeter). For GUS staining, plant tissues were soaked in 0.15 M phosphate buffer pH 7, vacuum-infiltrated and incubated for 1 hour at 37°C in GUS staining solution (0.5 mM 3-indolyl glucuronide, 0.15 M NaH_2_PO_4_ pH 7, 2 mM K_3_Fe(CN)_6_, 2 mM K_4_Fe(CN)_6_, 0.05% Triton ×100).

### Extraction and Determination of Sugars and Organic Acids

Slices from 8 daa tomato fruit were incubated in the various conditions as described for biolistic transient expression assays, weighed, frozen in liquid nitrogen and ground in a mortar. Briefly, soluble sugars and starch were extracted using alcoholic extraction method and starch converted to glucose as previously described [42]. Soluble sugars were then measured using a MR 5000 microplate reader (Dynatech) microassay. Citric and malic acids were extracted as previously described [12] and enzymatically measured following instructions of the Boehringer’s kit adapted for MR5000 reader micro assay.

## Results and Discussion

### Features of the SlPPC2 Promoter

Plant PEPCs show a highly conserved structure and amino acid sequence [16]. Like most other plant PEPC genes, the *SlPPC2* gene is formed of 10 exons interrupted by nine introns located at conserved positions (Figure S1). Comparison of *SlPPC2* genomic sequence and 5′ UTR sequence of *SlPPC2* cDNA also revealed the presence of an additional intron in the 5′ leader sequence of *SlPPC2*. Its location and size (200 bp) is close to that of the leader intron found in the well-studied C4 PEPC *ppcA1* gene from dicot *F. trinervia ppcA1* gene (177 bp), which is expressed in mesophyll cells and fulfils very different roles [12], [21], [23], [43].

**Figure 1:**
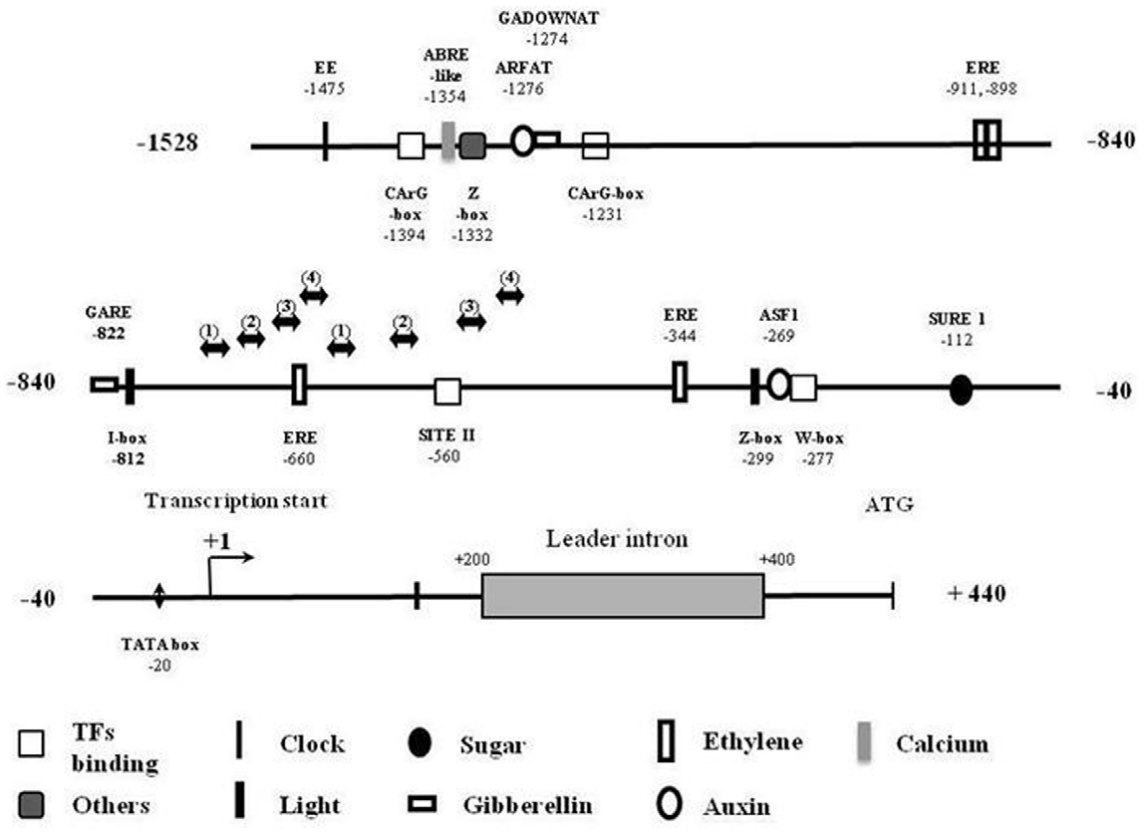
Localization of putative *cis*-acting elements in *SlPPC2* promoter sequence. Sequence was analyzed using PLACE and PlantCARE databases.

A *SlPPC2* promoter fragment including the 5′ untranslated region (UTR) of the gene (–1969 to −3 bp from the translation start site) was obtained by PCR amplification and restriction. The putative transcriptional start point determined by primer extension analysis was located 442 nucleotides upstream of the translational start codon ATG (Figure S2). A putative TATA box is located at nucleotide –20 relative to the transcriptional start point. Analysis with PLACE [31] and PlantCARE [32] unravelled putative *cis*-regulating elements known to play a role in the regulation of transcription. In addition, several motifs identified as binding sites for transcription factors (MADS domain factors, TCP, WRKY) were also found upstream of transcription start (Figure 1). Of particular interest is the −1500 to −900 region where motifs putatively involved in the binding of MADS domain protein (CArG box) [44] and in signalling pathways for auxin and brassinosteroid (ARFAT) [45], gibberellin (GADOWNAT and GARE) [46], [47], abscisic acid and calcium (ABRE-like motif) [48], [49] and ethylene (ERE) [50] were found. In plants, regulatory elements usually tend to be highly clustered in the vicinity of the core-promoter elements, but can also be found all along the promoter [51], [52]. In tomato, the distal 5′ flanking regions are crucial for the regulation of at least two genes, the ripening-associated tomato polygalacturonase (PG) and the E8 gene [53]–[55].

Several hormones play a prominent role in the regulation of early fruit growth. Among these are the auxins and brassinosteroids, which have a synergistic effect on cell elongation in plants [56], and may control the expansion of the fruit mesocarp cells in which *SlPPC2* is expressed [5], [12], [57], [58]. The fruit ripening hormone ethylene can also be implicated in early fruit growth in tomato, owing to its role in the control of endoreduplication and cell expansion in various plant species and organs [59]–[61]. Other elements identified in light or circadian-regulated genes are scattered along the *SlPPC2* promoter sequence (I box, GT1 element, Evening Element EE and Z box) [50], [62]–[65]. Though there is no evidence for light or circadian clock regulation of PEPC in fruit, the light involvement in the regulation of fruit development and metabolism is now well established [9] and recent results indicate that a sugar-metabolism gene, the LIN6 invertase, is regulated by diurnal rhythm in tomato fruit [66]. A SURE motif [67] and two G boxes separated by 17 bp including ACT [68] can also indicate the involvement of sugars in *SlPPC2* regulation. The search for fruit-specific elements identified in other plant species [69], [70] remained unsuccessful.

### The SlPPC2 Promoter Confers Proper Developmental Regulation in Developing Tomato Fruit

**Figure 2:**
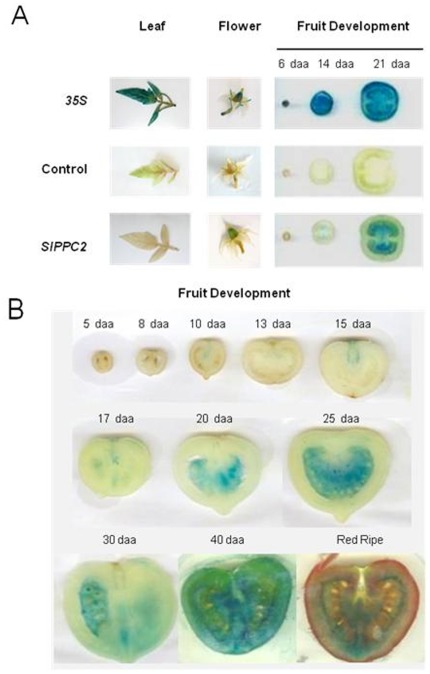
GUS activity in tomato (cv. «Ferum») stably transformed with *SlPPC2* promoter:GUS transgenes. (A) Representative images of GUS activity in seedlings, leaf, flower and 6, 14 and 21 days after anthesis (daa) tomato fruit with the 35S:GUS and *SlPPC2* promoter:GUS constructs. (B) Representative images of GUS activity in «Ferum» fruits at the various stages of fruit development (daa) as indicated.

The miniature MicroTom tomato previously used to monitor *SlPPC2* promoter activity in tomato [10] is likely mutated in the brassinosteroid *dwarf* gene and may thus display altered hormonal and developmental regulations. To investigate whether the organ-specificity and developmental patterns observed in MicroTom were conserved in other tomato genotypes, transgenic tomato plants were generated with *SlPPC2* promoter:GUS or *SlPPC2* promoter:GFP-GUS transcriptional fusions, using two different tomato cultivars. The cultivars used were «Ferum», a cultivated greenhouse tomato variety with medium-sized fruits, and «Wva106», a cherry-type tomato well adapted to the study of early fruit development [14], [15], [71]. In «Ferum», the *SlPPC2* promoter:GUS primary transformants showed consistently (>10 independent transformants) GUS staining in expanding fruit tissues but exhibited no staining of young seedlings, leaves or flowers (except for faint staining of stamens) (Figure 2A). A representative GUS staining of T2 homozygous fruits (single copy insertion line) is shown in Figure 2B. Time-course analysis of *SlPPC2* promoter activity along fruit development indicated that *SlPPC2* promoter activity peaked between 25 and 40 daa, i.e. during the cell expansion phase which lasts from ∼10 to 40 daa in the «Ferum» cultivar [71]. During fruit development, the staining progressed from the placental tissue, which differentiates early, to the outer pericarp. No staining was observed during the early stages of cell division while residual GUS activity was seen in ripe fruit. In contrast, 35S-driven GUS activity was high in all plant organs and fruit stages analyzed. Similar results were obtained in the cherry tomato «WVa 106» using *SlPPC2* promoter:GFP-GUS fusion (pKGWFS7 vector, data not shown). These results are consistent with the pattern of *SlPPC2* transcript accumulation in the plant [12] and with previous results obtained in MicroTom transgenics [10], thereby confirming that the *SlPPC2* promoter is specifically active in the fruit during the cell expansion phase.

**Figure 3:**
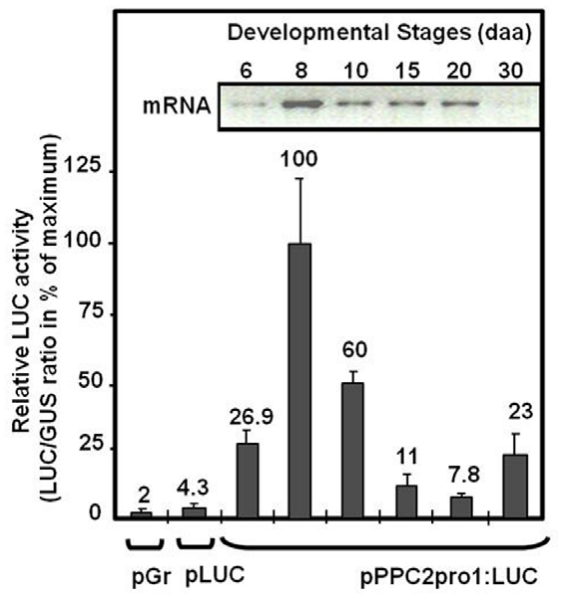
Transient reporter-gene expression analysis of the *SlPPC2* promoter in developing tomato fruit («WVa 106» cherry). Fruit slices at the indicated stages of development from 6 to 30 days after anthesis (daa) were transformed by biolistic with a 35S:GUS plasmid co-delivered with a promoter:LUC fusion plasmid (promoter:LUC construct pPPC2pro1:LUC) that included the nucleotides –1528 to +439 of *SlPPC2* (with respect to the transcription start site). The pGr (plasmid alone) and pLUC (promoterless LUC construct) plasmids were used as negative controls to transform 8 daa fruit slices. Data were normalized using the 35S:GUS construct as internal standard and are expressed as % of maximum activity (8 daa fruit). The mean values and SE of 6 to 12 independent transformations are shown. Insert represents the RT-PCR analysis of *SlPPC2* expression during wild-type tomato fruit development.

In order to analyse the regulation of *SlPPC2* promoter by various metabolites and hormones in developing fruit, we next used transient expression assays. We preferred this technique over the use of whole transgenic fruits expressing GUS for several reasons. Because tomato fruit is a bulky organ, the penetration and transport of hormones and metabolites in the various tissues from whole transgenic fruits is very difficult to control. This may therefore strongly bias the results and affect their reproducibility. In contrast, the method of biolistic transformation of osmotically-treated tomato fruit tissues developed by Baum and co-authors [36], allows quantitative, systematic and reproducible measurements in fruit tissues. In this method, the use of luciferase as reporter gene allows studying the fine control of promoter activity whereas using GUS as reference allows normalization of the data and therefore comparison between multiple experiments. In a first step, we tested this technique by fusing 1966 bp of the 5′ flanking regions of the *SlPPC2* gene (including 439 bp of the 5′ UTR and leader intron) to LUC (firefly Luciferase) reporter gene and by examining its expression in developing tomato fruit. Adaptation of this protocol to our conditions (see Materials and Methods) led to a consistent 35S promoter-driven luciferase activity that was about 600-fold over background activity in young green fruit from the “Wva106” cultivar (data not shown). Changing mannitol concentration in the incubation medium from 12% to 4% led to a further increase of 1.5 fold in the promoter activity. To take into account the possible light or circadian clock regulation of *SlPPC2* (see above), all experiments were conducted with fruits collected early in the morning at the same time. Under these conditions, tissues from tomato fruit at various stages of development (from 6 daa to 30 daa mature green stage) were co-transformed with the pPPC2pro1:LUC (*SlPPC2* promoter-LUC construct) and 35S:GUS constructs as internal controls. Results indicated that full-length *SlPPC2* promoter was sufficient to drive a high reporter gene activity in the developing fruit, with a notable expression from 6 to 15 daa and peaking at 8 daa, consistent with the timing of the cell expansion phase and changes in *SlPPC2* transcript abundance level in «WVA 106» fruit (Figure 3). Therefore, the 1966 bp of the 5′ flanking regions of the *SlPPC2* gene studied contains all the information necessary to confer proper developmental regulation in tomato fruit.

### Deletion of the Leader Intron Increases SlPPC2 Promoter Activity but Affects its Developmental Pattern

**Figure 4:**
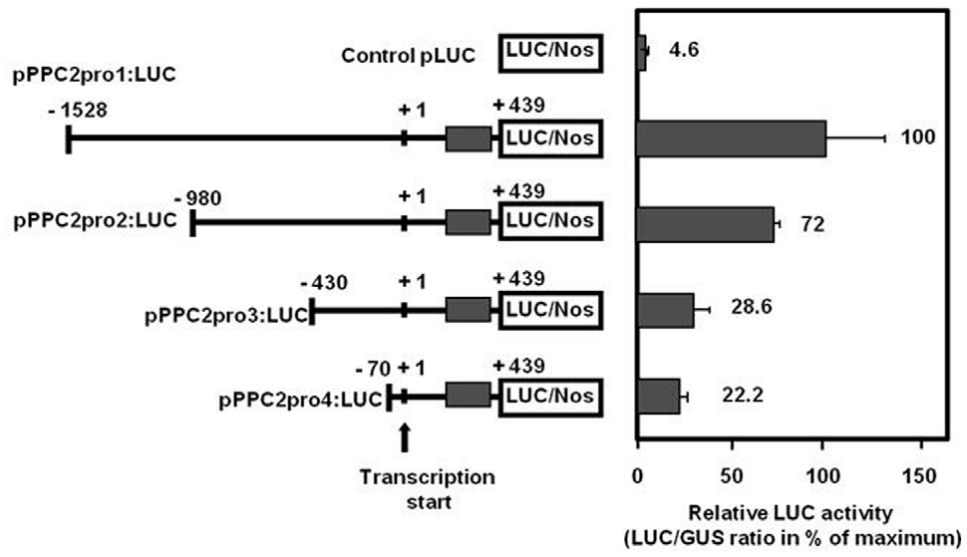
Transient reporter-gene expression analysis of *SlPPC2* promoter deletions in 8 daa tomato fruit. Slices from 8 daa fruit («WVa 106» cherry) were transformed by biolistic with a 35S:GUS plasmid co-delivered with *SlPPC2* promoter:LUC fusion plasmids (pPPC2pro1-4:LUC construct; sizes in nucleotides from the transcription start indicated; grey box indicates leader intron).The pLUC plasmid (promoterless LUC construct) was used as a negative control. Data were normalized using the 35S:GUS construct as internal standard and are expressed as % of maximum activity (pPPC2pro1:LUC construct). The mean values and SE of 6 to 12 independent transformations are shown.

To further analyze the role of the *SlPPC2* 5′ flanking regions and of the leader intron in the regulation of *SlPPC2* gene expression, a set of 5′ deletions was produced (Figure 4) and their expression was analyzed in 8 daa fruit, when *SlPPC2* full-length promoter activity (pPPC2pro1:LUC construct) is maximum (Figure 3). Deletion to position −980 (pPPC2pro2:LUC) reduced the activity relative to pPPC2pro1:LUC by about 28% in 8 daa fruit. Additional deletions to positions −430 and −70 further reduced the activity relative to pPPC2pro1:LUC by about 71% and 78%, respectively. Control (pLUC) only showed a marginal luciferase activity (4.6% of the activity of pPPC2pro1:LUC at 8 daa). These data suggest that the major *cis*-acting elements responsible for high level of *SlPPC2* promoter activity in young fruit (8 daa) are located between positions −1528 to –430, a region which is particularly rich in putative regulatory elements (Figure 1).

**Figure 5:**
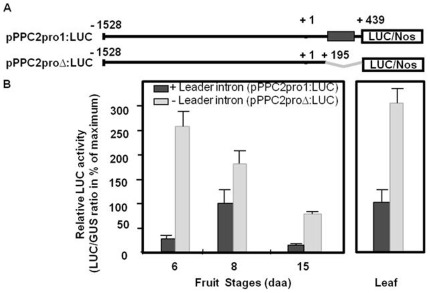
Transient reporter-gene expression analysis of the *SlPPC2* promoter deleted from its leader intron in developing tomato fruit and in leaf. (A) Details of the constructs. (B) Young leaf discs and slices of tomato fruit («WVa 106» cherry) at the indicated stages of development were transformed by biolistic with the –1528 to +439 construct (pPPC2pro1:LUC, in black) or with the –1528 to +195 construct (pPPC2proΔ:LUC, in grey) as indicated in Figure 1. Data are expressed as % of the pPPC2pro1:LUC activity at 8 daa. The mean values and SE of 10 independent transformations are shown.

To test whether the 200-bp intron located in the 5′ UTR is important for the control of the developmental expression, we deleted the region spanning from +195 to +439 (pPPC2proΔ:LUC construct), which comprises both the leader intron and the 5′ UTR between the leader intron and the start codon. Deletion of the leader intron enhanced the transcriptional activity of the promoter by 1.8 to 9.6-fold, depending on the developmental stage of the fruit, and led to a loss of its proper regulation during fruit development (Figure 5). Contrary to the activity of the full length promoter (pPPC2pro1:LUC construct) and to the level of *SlPPC2* transcripts (Figure 3), the activity of pPPC2proΔ:LUC was much higher at 6 daa, i.e. in mitotic cells, than in tissues undergoing cell differentiation and expansion. In this context, these data provide clear evidence that the first intron functions as a negative regulatory element that contributes to the developmental regulation of *SlPPC2* expression in the fruit.

Both positive and negative roles for leader introns have been demonstrated in several plant genes including the sucrose synthase gene *SUS3* [72] and the *Arabidopsis* cytochrome C oxidase gene *COX5C* in which the leader intron is essential to direct high-level and tissue-specific expression [73]. In contrast, recent work on the *F. trinervia* C4 isoform of PEPC suggested that the leader intron in this gene is not essential for achieving high mesophyll-specific expression [74]. A growing number of plant expression studies have also revealed that the presence of a leader intron within the 5′UTR may affect not only transcription but also post-transcriptional processes [73], [75]. Regardless of the control level of LUC activity exerted by the leader intron, the main conclusion is that the full-length promoter (including leader intron) is necessary to deliver mRNA/protein to fruit cells specifically during the cell expansion stage of tomato fruit development.

Surprisingly, in transient expression assays, fruit-specificity was lost since pPPC2pro1:LUC and pPPC2proΔ:LUC activities were similar in leaf and in 6 daa or 8 daa fruit, respectively (Figure 5). This was also true for the various deletion constructs tested (data not shown). The above findings suggest that chromatin integration is essential to confer an appropriate pattern of expression in the plant, as previously found for the tomato fruit *RBCS3A* promoter [76].

### Hormonal Regulation of the SlPPC2 Promoter

**Figure 6:**
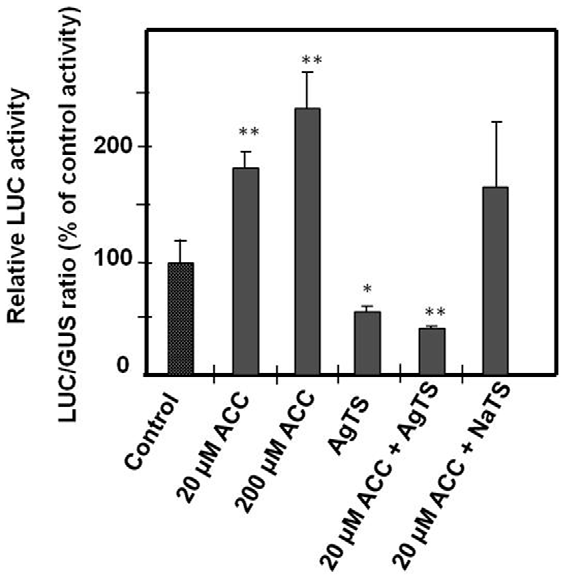
Influence of ethylene (ACC) on *SlPPC2* promoter activity. Fruit slices from 8 daa fruit («WVa 106» cherry) were transformed by biolistic with a 35S:GUS plasmid co-delivered with *SlPPC2* promoter:LUC fusion pPPC2pro1:LUC plasmid and incubated for 20 h on CPW4 medium supplemented with 1-aminocyclopropane-1-carboxylic acid (ACC, 20 µM and 200 µM), silver thiosulfate (AgTS), or ACC (20 µM) plus AgTS or NaTS. Control was CPW4 medium. Data were normalized using the 35S:GUS construct as internal standard and are expressed as % of the control. The mean values and SE of 12 independent transformations are shown. * indicates a significant statistical difference using a Student’s *t*-test (*: *P*<0.05; **: *P*<0.001).

Hormones are known regulators of fruit set and early fruit development [13] and several putative hormone responsive elements were identified in the *SlPPC2* 5′ flanking region by *in silico* analysis (Figure 1). Therefore, the effects of auxins (2,4-D), cytokinins (kinetin), gibberellins (GA3), abscisic acid (ABA) and ethylene precursor 1-aminocyclopropane-1-carboxylic acid (ACC) on full-length *SlPPC2* promoter activity were investigated using biolistic transient expression assay. Kinetin (5 µM), GA3 (5 µM) and ABA (50 µM) did not display any significant effect on *SlPPC2* promoter activity (data not shown). By contrast, the synthetic auxin 2,4-D significantly increased the *SlPPC2* transcriptional activity when applied at 50 µM (data not shown), whereas the 2,4-D non-functional analog 2,3-D failed to trigger any change in *SlPPC2* promoter activity, at the same concentration. Auxin plays a major role in early fruit development in addition to its well known effect on fruit set [56], [77]–[79]. However, 2,4-D is usually physiologically active at much lower concentrations (<5 µM), suggesting that the 2,4-D effect on *SlPPC2* promoter activity is indirect. In contrast, the ethylene precursor ACC, fed to pericarp discs, had a strong and significant effect on *SlPPC2* transcriptional activity (Figure 6). Notably, ACC significantly increased *SlPPC2* promoter activity at 20 µM and enhanced it by two-fold at 200 µM (Figure 6). In the presence of silver thiosulfate (AgTS), a known inhibitor of ethylene action [39], promoter activity was significantly reduced, even in the presence of ACC at 20 µM. In contrast, its inactive AgTS analogue failed to inhibit the action of ethylene produced by 20 µM ACC. Though ethylene is much better known for its coordination of fruit ripening [80], this hormone may control endoreduplication and cell expansion in various plant species and organs [59]–[61]. It is therefore likely that ethylene is involved in the regulation of the cell expansion phase in early developing fruit, as suggested by the analysis of the auxin mutant *diageotropica* [78].

We further investigated whether the ethylene-modulated changes in *SlPPC2* transcriptional activity were accompanied by variations in organic acid content in tomato fruit tissues. Significant effects were observed with 200 µM ACC, which increased L-citric acid and L-malic acid contents by 50% and 60%, respectively (Table 1). Conversely, addition of AgTS to pericarp discs fed with 20 µM ACC resulted in a slight but significant reduction in L-citric acid content of about 35% (Table 1). These results are consistent with transgenic experiments in which PEPC overexpression in tobacco redirects the metabolic flow, in particular towards malate synthesis [81], [82].

**Table 1:** Malic and citric acid contents of 8 daa tomato fruit slices.

Treatment	Malic acidnmol/gFW)	Citric acid(nmol/gFW)
Control (CPW4)ACC (20 µM)ACC (200 µM)ACC (20 µM)+AgTS	22.1±1.827.4±6.335.2±6.3*17.8±3.4	32.2±1.441.6±8.248.6±5.2*21.1±2.4*

Fruit slices were incubated on CPW4 medium (Control) and subjected to various treatments as indicated. The mean values and SE of 6 independent transformations are shown. Asterisk (*) indicates a significant statistical difference using a Student’s *t*-test (*P*<0.05).

### Metabolic Regulation of the SlPPC2 Promoter

**Figure 7:**
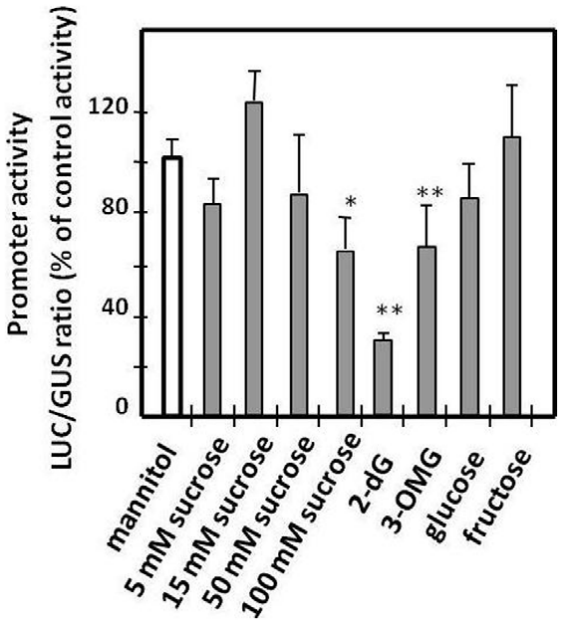
Influence of sugars on *SlPPC2* promoter activity. Fruit slices from 8 daa fruit («WVa 106» cherry) were transformed by biolistic with a 35S:GUS plasmid co-delivered with *SlPPC2* promoter:LUC fusion pPPC2pro1:LUC plasmid and incubated for 20 h on CPW4 medium supplemented with sucrose concentrations ranging from 5 mM to 100 mM as indicated, 3-O methylglucose (3-OMG, 50 mM), 2-deoxyglucose (2-dG, 50 mM), glucose (50 mM) or fructose (50 mM). Control was CPW4 medium supplemented with mannitol (50 mM). Data were normalized using the 35S:GUS construct as internal standard and are expressed as % of the control. The mean values and SE of 12 independent transformations are shown. * indicates a significant statistical difference using a Student’s *t*-test (*: *P*<0.05; **: *P*<0.001).

Various metabolites such as sugar hexoses may regulate PEPC transcription [25]. In order to investigate the possible regulation of *SlPPC2* by sugars, the activity of the *SlPPC2* full-length promoter was determined on bombarded 8 daa tomato fruit slices incubated with various concentrations of sucrose, glucose, fructose, the glucose analogs 2-deoxyglucose (2-dG) and 3-O-methylglucose (3-OMG), or mannitol as an osmotic control. We first controlled that the sugars were taken up by the fruit tissues by measuring the concentration in various metabolic compounds (sucrose, glucose, fructose, starch, malic and citric acids) in fruit discs at the end of the incubation period (see Figure S3). Sugars could effectively enter the fruit slices and were metabolized, as evidenced by the cleavage of sucrose to glucose and fructose, the interconversion of glucose and fructose and the synthesis of starch and organic acids further accumulated in fruit tissues. This indicated that fruit slices are suitable for studying the regulation of *SlPPC2* by sugars. While no significant alterations of *SlPPC2* promoter activity were observed in sugar-supplemented fruit tissues, our data showed that high sucrose concentration (100 mM) resulted in a signification reduction in promoter activity (Figure 7). However, because mannitol was used at 50 mM in the control, a possible osmotic effect of 100 mM sucrose on *SlPPC2* promoter activity cannot be excluded.

In contrast, *SlPPC2* promoter activity was reduced by about one-third when the glucose analog 3-O-methylglucose (3-OMG) was supplied at 50 mM to the tissues, and reduced by two-thirds when 2-deoxyglucose (2-dG) was supplied at the same concentration (Figure 7). Malic acid content was significantly reduced only in 2-dG supplied tissues, in which *SlPPC2* promoter activity was strongly affected, whereas sugar (sucrose, glucose and fructose) or starch contents were not significantly affected in the tissues supplied with either 3-OMG or 2-dG (Figure S3). Results obtained for 2-dG are consistent with previous observations showing that variation in PEPC transcription may lead to changes in malic acid content in plant [81], [82] and fruit tissues [83]. The 2-dG can be transported into the tomato fruit cells and phosphorylated by hexokinase, but the phosphorylated product 2-deoxyglucose 6-phosphate (2-dG-6p) cannot be further metabolized [84]. The 3-OMG is transported into the plant cells but is metabolized very slowly [85]. Thus, the repression caused by 3-OMG and 2-dG supports the hypothesis that sugar is required for regulation of *SlPPC2* expression in the fruit. However, we cannot rule out that this result is achieved through a more general effect on fruit metabolism.

Inorganic nitrogen (NO_3_^−^, NH_4_^+^) and transported forms of amino acids in the fruit (glutamine and asparagine) were also tested but no significant effect on *SlPPC2* promoter activity was detected. Thus, under the experimental conditions of the study, there is no conclusive evidence of the transcriptional control of *SlPPC2* by metabolites other than sugar in the fruit.

### Conclusion

This study demonstrated that a 1966 bp 5′ region of the *SlPPC2* fruit PEPC gene including −1528 bp of promoter region plus 439 bp of 5′ untranslated leader region is able to confer appropriate fruit-specificity and developmental expression in tomato fruit. Transient expression assays further showed that the deletion of an intron in the 5′ untranslated leader region leads to loss of proper developmental regulation, suggesting that leader intron acts as a negative regulatory element. Though no correlation was found between promoter activity and sugar metabolites, sugar signaling may modulate *SlPPC2* promoter activity, as indicated by the effects of 2-dG and 3-OMG. Noticeably, results indicate that *SlPPC2* may be regulated by the plant hormone ethylene. While auxin is known for its role in the regulation of fruit growth [73], [74], the implication of ethylene and of cross-talks between auxin and ethylene for controlling early stages of fruit development has been poorly studied. The enhancement of *SlPPC2* promoter activity by ethylene and the concomitant organic acid increase in fruit tissues are consistent with the hypothesis that the PEPC-mediated organic acid synthesis sustains osmotic potential to allow rapid fruit cell expansion [12], [28] and is under hormonal control in developing tomato fruit. In addition, this study opens the way for the use of *SlPPC2* promoter for the functional study of candidate genes in the fruit and for the biotechnological improvement of fruit sensorial and nutritional quality.

## Supporting Information

Figure S1 **Exon/intron organization of the tomato** ***SlPPC2*** **gene.** The tomato *SlPPC2* gene (GenBank accession No. AJ313434) was compared to the *Flaveria trinervia ppcA1* gene (Genbank accession No. AJ011844). Introns (grey boxes) are numbered from I to X and their sizes indicated above the diagrams.
(TIF)

Figure S2 **Determination of the transcription start point of the SlPPC2** ***gene*** **by primer extension analysis.** Lane PE shows the extension product obtained after reverse transcription using a *SlPPC2*-specific oligonucleotide primer. The band, indicated by an arrow, corresponds to a G located 442 nucleotides upstream from the ATG codon. The sequencing ladder was generated using the same primer on a cloned fragment of the *SlPPC2* genomic clone. Sequence upstream from the transcription start site is presented, showing location of putative TATA box.
(TIF)

Figure S3 **Carbohydrate content of tomato fruit slices incubated on medium supplemented with various sugars.** (A) Sucrose; (B) Glucose; (C) Fructose; (D) Starch; (E) Malic acid; (F) Citric acid. Eight (8) daa tomato fruit slices were incubated or not (no incubation) for 20 h on CPW4 medium containing 50 mM mannitol, 5 mM to 100 mM sucrose, 50 mM 3-OMG, 50 mM 2-dG, 50 mM glucose or 50 mM fructose. Data are means ± SE (n = 3).
(TIF)
