# Supplementary figures and images for: Regulation of the Fruit-Specific PEP Carboxylase SlPPC2 Promoter at Early Stages of Tomato Fruit Development
Source: PLoS One. 2012 May 17;7(5):e36795. doi: 10.1371/journal.pone.0036795 (PMC3355170; doi:10.1371/journal.pone.0036795)

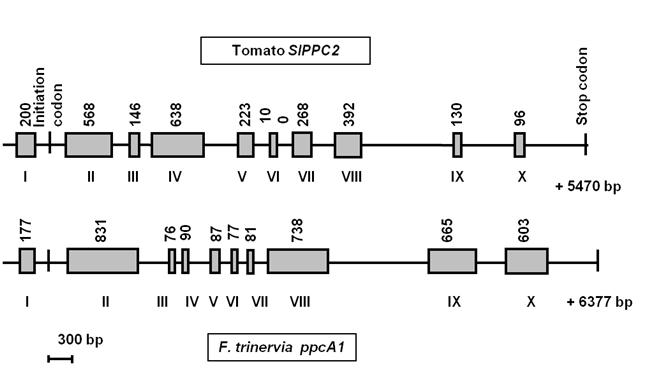

Supplement: Figure S1 — Exon/intron organization of the tomato SlPPC2 gene. The tomato SlPPC2 gene (GenBank accession No. AJ313434) was compared to the Flaveria trinervia ppcA1 gene (Genbank accession No. AJ011844). Introns (grey boxes) are numbered from I to X and their sizes indicated above the diagrams. (TIF) [file pone.0036795.s001.tif]

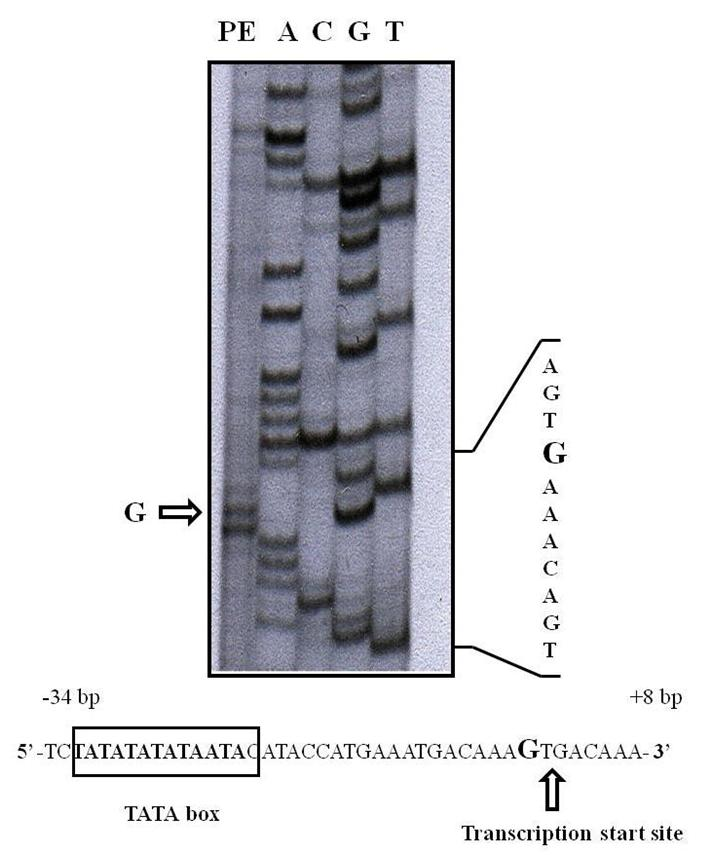

Supplement: Figure S2 — Determination of the transcription start point of the SlPPC2 gene by primer extension analysis. Lane PE shows the extension product obtained after reverse transcription using a SlPPC2-specific oligonucleotide primer. The band, indicated by an arrow, corresponds to a G located 442 nucleotides upstream from the ATG codon. The sequencing ladder was generated using the same primer on a cloned fragment of the SlPPC2 genomic clone. Sequence upstream from the transcription start site is presented, showing location of putative TATA box. (TIF) [file pone.0036795.s002.tif]

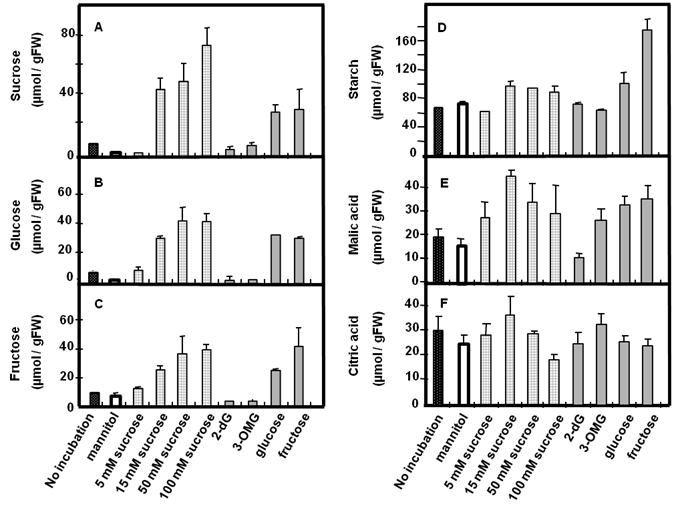

Supplement: Figure S3 — Carbohydrate content of tomato fruit slices incubated on medium supplemented with various sugars. (A) Sucrose; (B) Glucose; (C) Fructose; (D) Starch; (E) Malic acid; (F) Citric acid. Eight (8) daa tomato fruit slices were incubated or not (no incubation) for 20 h on CPW4 medium containing 50 mM mannitol, 5 mM to 100 mM sucrose, 50 mM 3-OMG, 50 mM 2-dG, 50 mM glucose or 50 mM fructose. Data are means ± SE (n = 3). (TIF) [file pone.0036795.s003.tif]
